# Decoding Gene Expression Signatures Underlying Vegetative to Inflorescence Meristem Transition in the Common Bean

**DOI:** 10.3390/ijms232314783

**Published:** 2022-11-26

**Authors:** Ana M. González, Ricardo Lebrón, Fernando J. Yuste-Lisbona, Cristina Gómez-Martín, Ana Ortiz-Atienza, Michael Hackenberg, José L. Oliver, Rafael Lozano, Marta Santalla

**Affiliations:** 1 Genética del Desarrollo de Plantas, Misión Biológica de Galicia-CSIC, P.O. Box 28, 36080 Pontevedra, Spain; 2 Centro de Investigación en Biotecnología Agroalimentaria (CIAIMBITAL), Universidad de Almería, 04120 Almería, Spain; 3 Departamento de Genética, Facultad de Ciencias & Laboratorio de Bioinformática, Centro de Investigación Biomédica, Universidad de Granada, 18071 Granada, Spain

**Keywords:** common bean, meristem, flowering, gene regulatory network

## Abstract

The tropical common bean (*Phaseolus vulgaris* L.) is an obligatory short-day plant that requires relaxation of the photoperiod to induce flowering. Similar to other crops, photoperiod-induced floral initiation depends on the differentiation and maintenance of meristems. In this study, the global changes in transcript expression profiles were analyzed in two meristematic tissues corresponding to the vegetative and inflorescence meristems of two genotypes with different sensitivities to photoperiods. A total of 3396 differentially expressed genes (DEGs) were identified, and 1271 and 1533 were found to be up-regulated and down-regulated, respectively, whereas 592 genes showed discordant expression patterns between both genotypes. *Arabidopsis* homologues of DEGs were identified, and most of them were not previously involved in *Arabidopsis* floral transition, suggesting an evolutionary divergence of the transcriptional regulatory networks of the flowering process of both species. However, some genes belonging to the photoperiod and flower development pathways with evolutionarily conserved transcriptional profiles have been found. In addition, the flower meristem identity genes *APETALA1* and *LEAFY*, as well as *CONSTANS-LIKE 5*, were identified as markers to distinguish between the vegetative and reproductive stages. Our data also indicated that the down-regulation of the photoperiodic genes seems to be directly associated with promoting floral transition under inductive short-day lengths. These findings provide valuable insight into the molecular factors that underlie meristematic development and contribute to understanding the photoperiod adaptation in the common bean.

## 1. Introduction

Sexual reproduction in plants is fully dependent on a combination of favorable environmental circumstances and endogenous developmental cues. Our understanding of such floral transition signaling pathways is largely restricted to the model plant *Arabidopsis*, and they remain elusive in crops of agricultural relevance. The common bean (*Phaseolus vulgaris* L.) is likely the most significant grain legume for human consumption globally since it is a staple meal in many regions of the developing world, supplying essential amino acids and nutrients as well as complex carbohydrates. As the common bean progresses from the vegetative to the reproductive phase of development, it experiences significant transformations associated with the relative allocation of photoassimilates and nutrients and by changes in the morphology of different plant organs over time [1]. This transition time is key in defining both adaptability and fitness, and a vital factor that determines crop productivity [2,3]. Therefore, plants have evolved genetic and molecular networks integrating various environmental cues (photoperiod, vernalization, temperature, and light) with endogenous signals (age, stress, and hormonal state) to flower under optimal conditions [4]. The photoperiod is one of the essential environmental triggers and the common bean, as a short-day (SD) plant blooms later when grown in latitudes with longer summer daylength [5,6,7]. The physiological reproductive variability has been previously studied in the common bean [1,8], although little is known about the genetic regulation of floral transition and the development network of its reproductive meristems.

The central developmental event in the flowering onset is the transition from a vegetative meristem (VM) that generates leaves, branches, and stems into an inflorescence meristem (IM) that forms the inflorescence branch, which has flowers or branches depending on its architecture [9]. Our knowledge about the gene regulatory networks that control the IM development is mostly based on studies in the long-day (LD) plant *Arabidopsis thaliana* [10], although new genetic floral transition models are being provided according to the diversity of inflorescence architectures in different crops [11]. Flowering is regulated by an integrated network of several genetic pathways in *Arabidopsis* [12,13,14,15,16,17]. The outcomes of these pathways ultimately converge into a subset of genes, commonly known as floral integrators, including *FLOWERING LOCUS T* (*FT*), *SUPPRESSOR OF OVEREXPRESSION OF CONSTANS1/AGAMOUS-LIKE 20* (*SOC1/AGL20*), and *LEAFY* (*LFY*), that coordinate the IM developmental program [18]. Although the orthologues of the *Arabidopsis* flowering-time genes and their targets are well conserved across many flowering species [19], the regulation of these genes and the functional relationships among their gene products may differ significantly from those of *Arabidopsis*.

*Arabidopsis* and legume crops belong to the rosid clade, and even though most genes and gene families described in *Arabidopsis* are to some extent conserved in legumes, numerous gene duplication, loss-of-function, and neofunctionalization events occurred after the divergence of both lineages [20,21,22,23,24,25]. Molecular flowering studies in legumes have mostly focused on the SD soybean (*Glycine max* L.) and LD pea (*Pisum sativum* L.) crops [22,25], and to a minor extent on temperate *Medicago truncatula* and *Lotus japonicus* species [22,26]. Given the key role of the *FLOWERING LOCUS C* (*FLC*) gene in *Arabidopsis* floral transition regulation, the lack of the legume *FLC* orthologue is perhaps the most striking difference between these species [27], although genes involved in *FLC* inhibition such as *FLOWERING CONTROL LOCUS A* (*FCA*), *FLOWERING LOCUS D* (*FLD*), *FLOWERING LOCUS K* (*FLK*), *FPA*, *FLOWERING LOCUS VE* (*FVE*), *FY*, and *LUMINIDEPENDENS* (*LD*) have orthologues in *M. truncatula* and pea [20]. The legume *LFY* orthologue plays a role in flowering initiation similar to the gene reported in *Arabidopsis*, but also has a distinctive function in leaf development [28]. The *GIGANTEA* (*GI*), *EARLY FLOWERING* (*ELF4*), and *ELF3* orthologues play the same role as their *Arabidopsis* counterparts in the regulation of *FT* genes and photoperiod responsiveness during floral transition in pea, lentil (*Lens culinaris* M.), soybean, and chickpea (*Cicer arietinum* L.) [29,30,31,32,33], indicating that photoperiod and circadian clock pathways are strongly interconnected. *PHYTOCHROME A* (*PHYA*) and *CONSTANS-LIKE* (*COL*) homologues are found in legumes [24] and play a central and conserved role in photoperiod sensing in common beans [34,35], although it is unclear whether this function is preserved across all legume species [19,36,37]. Even though the roles of a few genes involved in common bean flowering regulation have been identified, almost nothing is known about the genetic regulation of meristem activity involved in the transition from VM to IM identity, as well as in the initiation of flowering because of the response to a photoperiodic environment. Here, we provide valuable insights towards this goal by characterizing genome-wide expression patterns that drive the transition from VM to IM in a photoperiod-insensitive cultivar and a photoperiod-sensitive wild accession. Thus, we identified those genes that are actively transcribed or repressed during flowering transition, which have allowed us to infer their involvement in the flowering onset of the common bean.

## 2. Results

### 2.1. Morphological Developmental Changes Occurring during Inflorescence Meristem Differentiation

To characterize the sequential meristem developmental progression of the common bean, two contrasting genotypes at floral transition under LD conditions (16 h light) were further studied at the transcriptional level. PHA0595 is an early-flowering bean cultivar and PHA1037 is a landrace with a strong photoperiod response similar to wild forms under LD conditions. Owing to the flowering variability that occurs under LDs, where PHA1037 is non-flowering, meristems were collected under inductive SD conditions (8 h of light). Even in SD conditions, PHA1037 exhibited a delay in flowering initiation and longer flowering period, higher number of nodes and internode length compared to PHA0595 [38].

**Figure 1:**
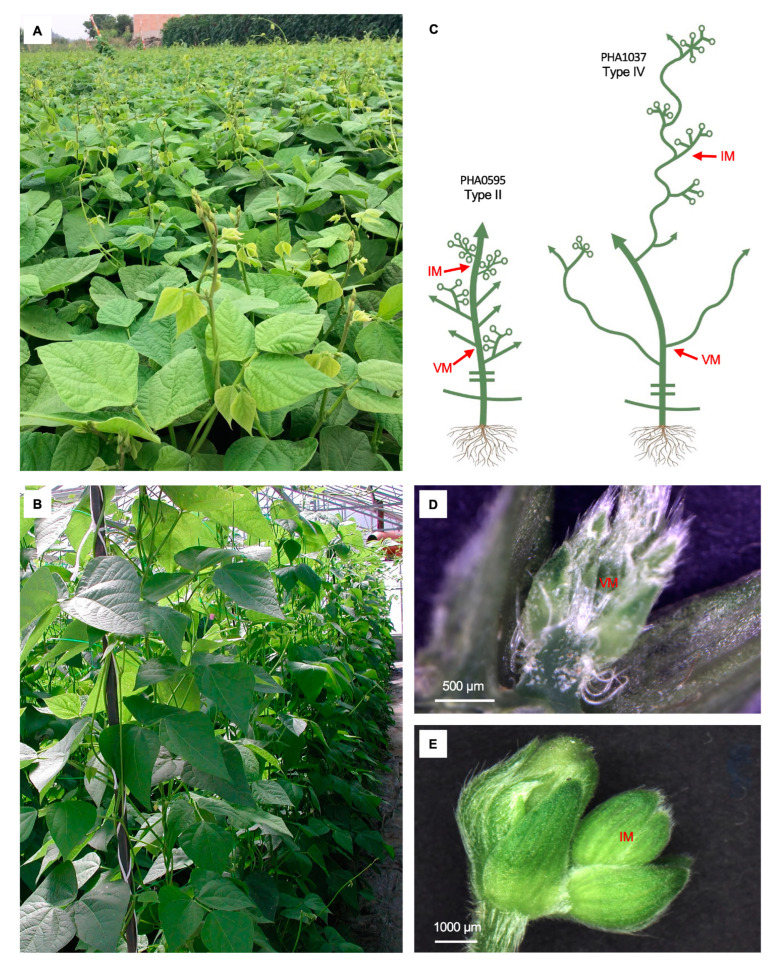
Morphological characteristics of the inflorescence differentiation process in common bean. Images of PHA0595 (**A**) and PHA1037 (**B**) genotypes representative of II and IV indeterminate growth habit types, and their plant architecture diagrams (**C**), respectively. In diagrams, arrows indicate indeterminate growth and circles indicate flowers. Photos of a vegetative axillary meristem (VM, (**D**)) and an inflorescence meristem (IM, (**E**)).

Both studied genotypes have an indeterminate growth habit, where the shoot apical meristem remains vegetative; it continues to develop forming nodes and internodes once the plant reaches the reproductive stage (Figure 1A,B). Therefore, the inflorescences appear at the axils of the branches and/or the trifoliate leaves as part of an axillary complex, which can simultaneously show vegetative and reproductive developments [39]. Different indeterminate II and IV archetypes are presented in PHA0595 and PHA1037 (Figure 1A–C), respectively. Plants with a type II growth habit develop a vegetative terminal bud on the main stem and branches, which are typically strong and upright [39], although some climbing ability can occur (Figure 1A–C). The main stem of the PHA1037 plants with an IV growth habit presents a height of approximately 20 nodes, whereas that of PHA0595 type II is generally approximately 12 nodes; its climbing ability appears from the first trifoliate leaf, and the stem and branches are weak and excessively long, possessing a strong ability to climb (Figure 1B,C).

Based on the morphological features of the inflorescence differentiation in the common bean, samples for transcriptome sequencing were collected at the undifferentiated axillary VM and IM stages (Figure 1D,E). The VM samples were collected at R5 pre-flowering stage [39], which corresponded to 41 and 48 d after germination in PHA0595 and PHA1037, respectively. At this stage, VMs were positioned in the incipient branch (Figure 1D), and they exhibited a flatted and narrow morphology, yellowish green color, and a matte texture with scale hairs. At the early inflorescence differentiation stage, IM samples were collected at 48 and 54 d after germination (R6 flowering stage) [39] in PHA0595 and PHA1037, respectively. The basal region of the IMs exhibited a broad and spherical shape with yellowish brown hairs on the outer surface, and the spathe-like bracts began to be stratified (Figure 1E).

### 2.2. Gene Expression Changes upon Differentiation to Inflorescence Meristem

To uncover transcriptomic changes related to flowering under SD photoperiod induction in the common bean, the VM and IM of two contrasting genotypes (PHA1037 and PHA0595) were further characterized using RNA-seq. Three biological replicates were analyzed for each genotype and each developmental stage. Our analyses were aimed at identifying those differentially expressed genes (DEGs) involved in the flowering process regardless of the origin of the genotype. The Euclidean pairwise-distance analysis was used to evaluate the consistency of biological replicates, confirming that all replicate data sets were highly consistent, as well as the distinctiveness of the developmental stage (Figure S1; Table S1).

**Figure 2:**
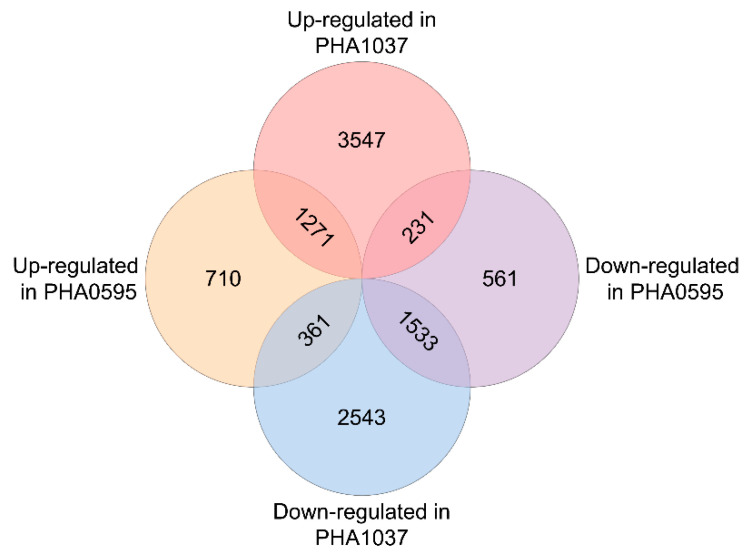
Venn diagram for differentially expressed gene sets in PHA1037 and PHA0595. Details of the differentially expressed genes can be found in Tables S2–S8.

Differential expression analysis revealed 5049 up-regulated and 4437 down-regulated genes in IM with respect to VM in the PHA1037 genotype (Tables S2 and S3; Figure S2). In the PHA0595 genotype, 2342 up-regulated and 2325 down-regulated genes were detected (Tables S4 and S5; Figure S3), indicating more pronounced transcriptomic differences between both types of meristems in the PHA1037 genotype. When comparing differential expression genes between PHA1037 and PHA0595 genotypes, 1271 up-regulated and 1533 down-regulated genes were determined to be common to both genotypes, whereas 592 genes exhibited discordant expression patterns (Figure 2; Tables S6–S8). These 592 genes with different expression profiles in each genotype are referred to as discordant genes. Among these genes, 231 were up-regulated in PHA1037 and down-regulated in PHA0595, and 361 were down-regulated in PHA1037 and up-regulated in PHA0595.

### 2.3. Biological Processes and Pathways Affected by Inflorescence Meristem Development

To better understand the biological processes involved in inflorescence meristem development, a Gene Ontology (GO) enrichment analysis was performed for up- and down-regulated genes common to both genotypes, as well as for discordant genes. As the functional annotation of *P. vulgaris* is poorly developed, the *Arabidopsis* homologous genes were used for this purpose. Homologous genes were assigned for 963 up- and 1326 down-regulated genes common to both genotypes and 467 discordant genes (Tables S9–S11). Among these genes, 44 enriched GO terms (organized into seven groups) were assigned to 210 up-regulated genes, 47 enriched terms (in 16 groups) to 294 down-regulated genes, and six enriched terms (in five groups) to 31 discordant genes (Tables S12–S14).

**Figure 3:**
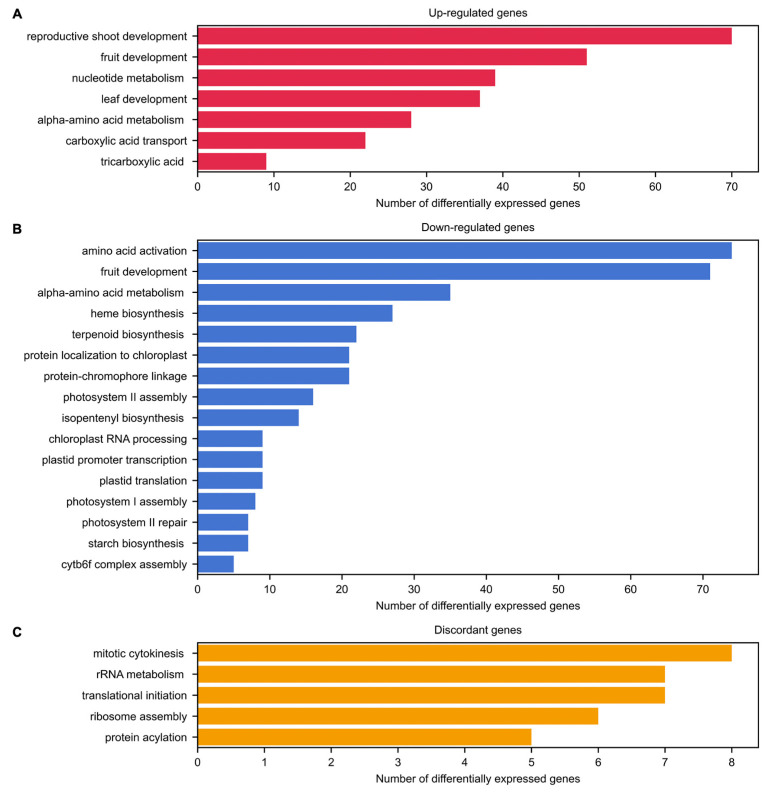
Biological processes in which differentially expressed genes are involved. The categories of GO terms for biological processes that are over-represented in each set of differentially expressed genes are the following: common up-regulated (**A**) and down-regulated (**B**) genes, and discordant genes (**C**), in the inflorescence meristem (IM) with respect to the vegetative meristem (VM) in PHA1037 and PHA0595. Details of this ontological annotation can be found in Tables S12–S14.

Most of the up-regulated genes were associated with GO terms of reproductive shoot and fruit development GO terms (Figure 3A; Table S12). Genes related to amino acid and carboxylic/tricarboxylic acid metabolism were also detected, which are involved in floral development [40]. Some of the up-regulated genes were also associated with leaf development, possibly as inhibitors of this process, such as the homologous to *AGAMOUS* genes (*AG*; *Phvul.006G169600* and *Phvul.002G243200*), previously described as a suppressor of the leaf development program in *Arabidopsis* emerging floral primordia [41]. Considering that the same gene can be associated with more than one biological process GO term, some genes related to leaf development were also involved in the development of the reproductive shoot, fruit development and tricarboxylic acid transport (Figure S4). Among the down-regulated genes, amino acid activation and fruit development GO terms were mainly overrepresented, with some genes engaged in both processes (Figure S5). Moreover, several genes were associated with transcription and translation in the plastids, as well as in the assembly and maintenance of photosystems (Figure 3B; Table S13). Changes in transcription and translation of plastids may be linked to the transition from chloroplast to chromoplasts [42]. The decrease in photosystem II activity during flower development also is known in other species such as grapevines, but a substantial decrease in photosystem I activity has not been previously observed [43]. Additionally, it is noteworthy that, among genes associated with enriched GO terms, there were 36 up-regulated and 13 down-regulated genes whose homologues in *Arabidopsis* have been previously reported as involved in any flowering-related pathway, according to the Flowering Interactive Database (FLOR-ID) [18].

**Figure 4:**
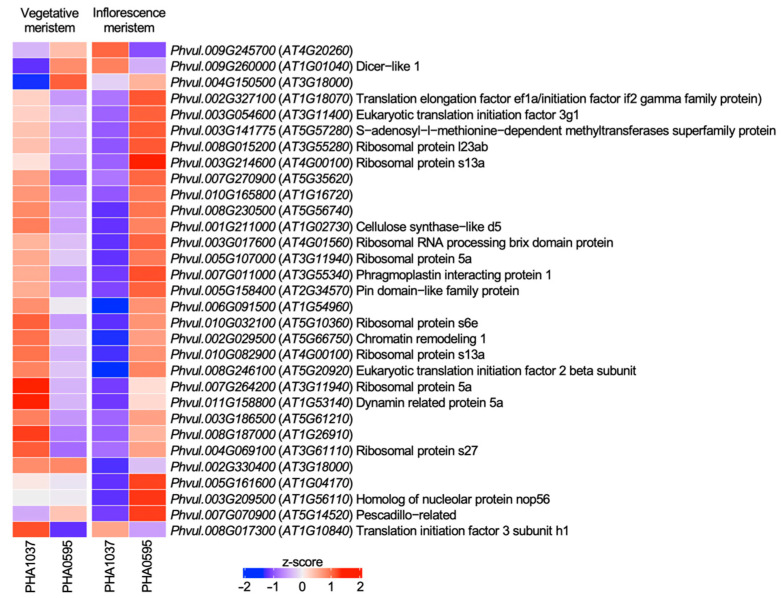
Transcription levels of discordant genes annotated with enriched GO terms showing opposite expression patterns in PHA1037 and PHA0595. For each gene, its expression levels have been represented as z-score using a color scale from blue (lowest level) to red (highest level). In each case, the identifiers of the common bean gene and its homologous gene in *Arabidopsis* are indicated (in brackets), as well as a description of the latter (when available). Details on these genes are given in Table S8.

The discordant genes between PHA1037 and PHA0595 genotypes were enriched for GO terms such as mitotic cytokinesis, rRNA metabolism, translational initiation, ribosome assembly and protein acetylation (Figure 3C; Table S14), with minimal overlap among the sets of genes involved in each process (Figure S6). Eight discordant genes were enriched for the mitotic cytokinesis process, which may be related to the different growth habits exhibited by each genotype (type II in PHA0595 and type IV in PHA1037). The expression pattern in both genotypes for the 31 discordant genes annotated with enriched GO terms is shown in Figure 4. Among these discordant genes, the only gene with a role in flowering that has been previously described is the homologue to *Arabidopsis DICER-LIKE 1* (*DCL1*; *Phvul.009G260000*), a ribonuclease III involved in RNA-mediated post-transcriptional gene silencing. In *Arabidopsis*, down-regulation of *DCL1* has been observed to produce later flowering times in both SD and LD photoperiod plants [44]. This result agrees with our observation that the expression of the homologous *DCL1* gene was down-regulated in PHA1037 but up-regulated in PHA0595 (Figure 4), as PHA1037 flowers later than PHA0595.

Moreover, the involvement of common and discordant DEGs between both genotypes in different biological pathways was also evaluated by performing a Kyoto Encyclopedia of Genes and Genomes (KEGG) pathway enrichment analysis. These KEGG pathways represent gene product interactions within a broad catalog of metabolic pathways, signaling pathways, and cellular components, among others. This analysis revealed 14, 12, and 1 enriched KEGG pathways associated with 119 up-regulated, 169 down-regulated, and 47 discordant genes, respectively (Tables S15–S17).

**Figure 5:**
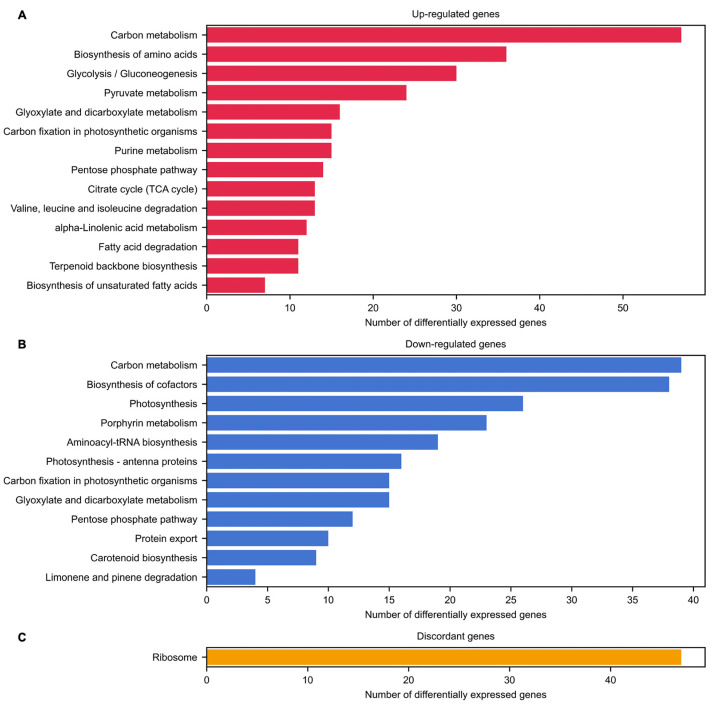
Biological pathways in which differentially expressed genes are involved. The KEGG pathways that are over-represented in each set of differentially expressed genes are the following: common up-regulated (**A**) and down-regulated (**B**) genes, and discordant genes (**C**), in the inflorescence meristem (IM) with respect to the vegetative meristem (VM) in PHA1037 and PHA0595. Details of this pathway annotation can be found in Tables S15–S17.

The findings of the GO term and KEGG pathway enrichment analyses were congruent with one another (Figure 3 and Figure 5). Most of the up-regulated genes were associated with carbon metabolism pathways, especially amino acids, nucleotides, and fatty acids metabolism (Figure 3A and Figure 5A; Tables S12 and S15). In contrast, most of the down-regulated genes were involved in photosynthetic processes and amino acid activation through the biosynthesis of aminoacyl-tRNAs (Figure 3B and Figure 5B; Tables S13 and S16). Lastly, genes with discordant expression patterns between PHA1037 and PHA0595 genotypes were associated with ribosome biosynthesis and assembly (Figure 3C and Figure 5C; Tables S14 and S17).

### 2.4. Evolutionarily Conserved Genes Involved in Flowering of the Common Bean

**Figure 6:**
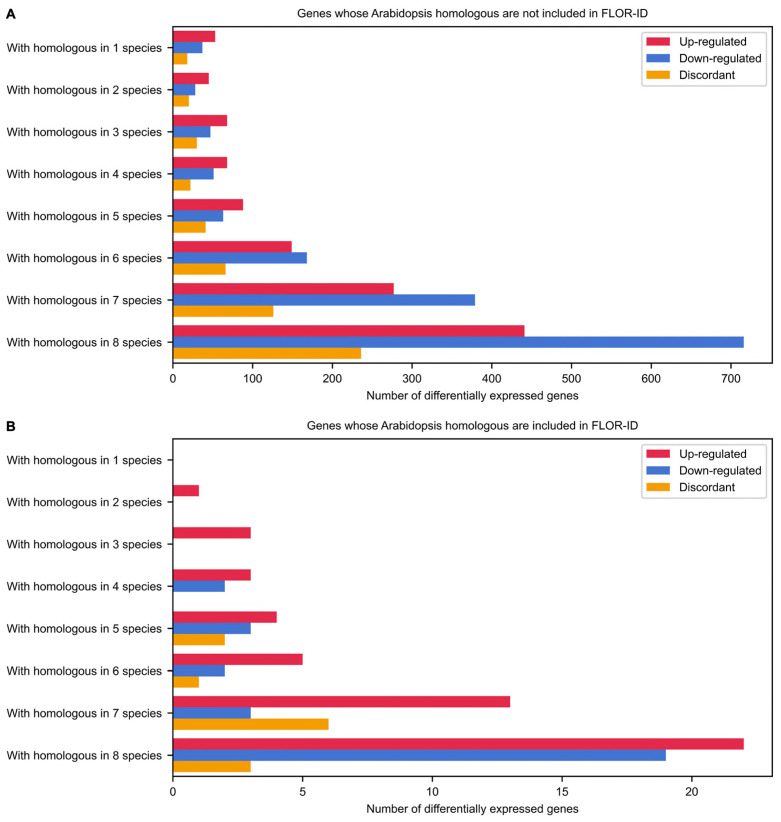
Evolutionarily conserved genes involved in inflorescence meristem development. Genes whose *Arabidopsis* homologous are not (**A**) and are included (**B**) in the FLOR-ID database as flowering-related genes [18]. The number of up-regulated and down-regulated genes common to both PHA1037 and PHA0595 cultivars are shown in red and blue, respectively. The number of genes with discordant expression profiles between PHA1037 and PHA0595 cultivars are plotted in orange. Details of these orthologous genes can be found in Tables S19 and S20.

With the aim of evaluating whether the common bean flowering genes have counterparts in other species, the degree of evolutionary conservation among DEGs common to both PHA1037 and PHA0595 genotypes was assessed for *Arabidopsis* and seven species of the Fabidae clade (Tables S18–S20), comprising the orders Rosales (*F. vesca*, *P. persica* and *M. domestica*), Cucurbitales (*C. sativus*), and Fabales (*G. max*, *M. truncatula* and *T. pratense*). The results revealed that 753 (59.24%) up-regulated, 1117 (72.86%) down-regulated, and 371 (62.67%) discordant genes have homologous genes in seven to eight of the evaluated species (Figure 6A), from which only 35, 22, and 9, respectively, were also included in the FLOR-ID *Arabidopsis* flowering database (Figure 6B; Table 1 and Table S21). Therefore, a high number of evolutionarily conserved DEGs were not previously described as involved in *Arabidopsis* flowering pathways. These findings suggest that there is a considerable evolutionary divergence between the flowering processes of *Arabidopsis* and that of the common bean, as well as a great number of genetic factors and biological pathways affecting the flowering transition of the common bean, which are still unknown. Thus, for example, within the set of up-regulated genes not included in FLOR-ID with homologous genes in the eight species assessed, the homologue of the tomato *COMPOUND INFLORESCENCE* (*Phvul.006G179900*) is worth noting, which encodes a WUSCHEL HOMEOBOX (WOX) transcription factor with expression that is specific to the IM promoting reproductive transition in tomato and other Solanaceae crops [45,46,47], but with no reported function in the *Arabidopsis* flowering regulatory network.

Among genes with homologous relationships in seven to eight species and included in FLOR-ID, we detected the homologues of the *Arabidopsis* flower meristem identity genes LFY (*Phvul.009G160900*) and *APETALA1* (*AP1*; *Phvul.003G281000*), which were up-regulated in the IM of both the PHA1037 and PHA0595 genotypes. In *Arabidopsis*, these transcription factors control the onset of flower development in a partially redundant manner [48]. Similarly, MADS box transcription factors were also found within the common up-regulated genes, such as the homologous of the B-, C-, and E-class floral organ identity genes *PISTILLATA* (*PI*; *Phvul.009G130000* and *Phvul.001G068200*), *AG* (*Phvul.006G169600* and *Phvul.002G243200*) and *SEPALLATA* (*SEP*; *Phvul.008G027900*, *Phvul.004G042300*, and *Phvul.L003446*), respectively. These genes, together with the A-class gene *AP1*, act in a combinatorial manner to control *Arabidopsis* floral organ specification [41].

In relation to the down-regulated genes, the homologue of *Arabidopsis CONSTANS-LIKE 5* (*COL5*; *Phvul.003G149000*) was detected, suggesting its role as a flowering inhibitor in the common bean, as previously described for its paralogous gene *CONSTANS-LIKE 2* (*COL2*; *Phvul.004G046601*) [35]. The homologue of *Arabidopsis PHYTOCHROME INTERACTING FACTOR 3* (*PIF3*; *Phvul.007G206000*) was also down-regulated and may act as a negative regulator of flowering. Previous studies reported that the silencing of these *Arabidopsis* homologous genes resulted in earlier flowering and increased expression levels of FT [49].

In *Arabidopsis*, *SOC1*/*AGL20* and *SHK1 BINDING PROTEIN 1* (*SKB1*) are known to be positive regulators of flowering [50,51]. In contrast, *ENHANCER OF AG-4 2* (*HUA2*) is a negative regulator of flowering and is involved in the regulation of *AG* mRNA processing [52]. Herein, the homologous genes of the *Arabidopsis SOC1/AGL20* (*Phvul.008G073800*) and *SKB1* (*Phvul.009G099900*) were down-regulated in PHA1037 and up-regulated in PHA0595, whereas the homologues of *Arabidopsis HUA2-LIKE 1* (*HULK1*; *Phvul.011G029450*) was up-regulated in PHA1037 and down-regulated in PHA0595. These findings are congruent with the early-flowering phenotype of PHA0595 when compared to that of PHA1037.

### 2.5. Candidate Marker Genes for Flowering Induction in the Common Bean

**Figure 7:**
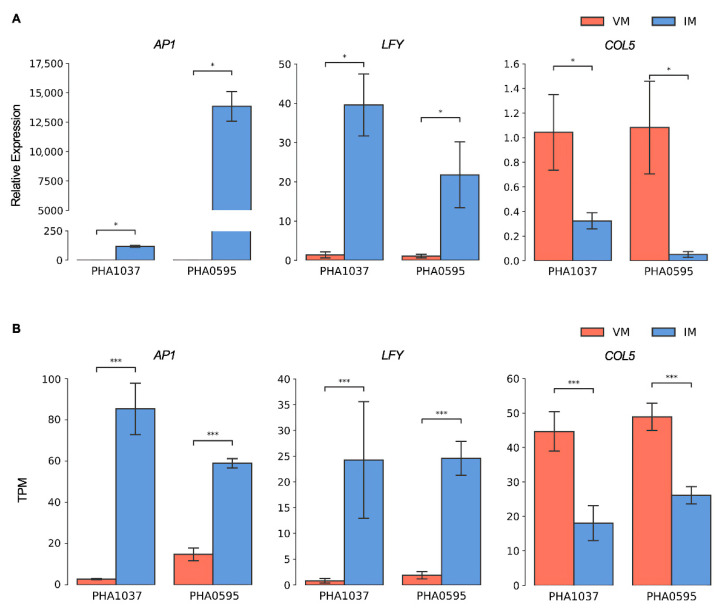
Validation of gene expression profiles obtained from RNA-seq by qRT-PCR. Expression patterns of the homologues of the *Arabidopsis APETALA1* (*AP1*; *Phvul.003G281000*), *LEAFY* (*LFY*; *Phvul.009G160900*) and *CONSTANS-LIKE 5* (*COL5*; *Phvul.003G149000*) genes examined by RT-qPCR (**A**) and RNA-seq (**B**) assays in vegetative axillary meristems (VM) and inflorescence meristems (IM) stages for the photoperiod-sensitive nuña PHA1037 accession and the early-flowering bean PHA0595 cultivar. Error bars represent the standard deviation from three biological replicates. Significant differences detected using Student’s t test are represented by asterisks: * *p* < 0.01; *** *p* < 0.0001.

To identify marker genes that could be useful tools to discriminate flowering times of different varieties through distinguishing between the vegetative and reproductive stages of meristems of the common bean, the expression patterns of three key homologous floral integrator genes were studied by using quantitative reverse transcription-polymerase chain reaction (qRT-PCR). Thus, we analyzed the expression of *LFY* (*Phvul.009G160900*) and its direct target *AP1* (*Phvul.003G281000*), which has been shown to be weakly expressed prior to the formation of the first flower in leaves but it is induced in floral meristems at later stages in *Arabidopsis* [53]. *AP1* is expressed in *Arabidopsis* primordia with a floral fate [54]. Our qRT-PCR and RNA-seq data showed that the *AP1* and *LFY* homologues of the common bean were upregulated in IM (Figure 7), which agrees with their reported function as flowering promoters in *Arabidopsis* [55], suggesting a conserved role of both meristem identity genes in the common bean.

In addition, we analyzed the expression of *COL5* (*Phvul.003G149000*), which is under circadian and diurnal regulation in *Arabidopsis* and is expressed in the vascular tissue of leaves and is present at low levels in flowers [56]. We found that the *COL5* homologue of the common bean was highly expressed in VM (Figure 7), whereas a lower expression was observed in IM, suggesting the role of *COL5* in the regulation of light-responsive flowering.

## 3. Discussion

**Figure 8:**
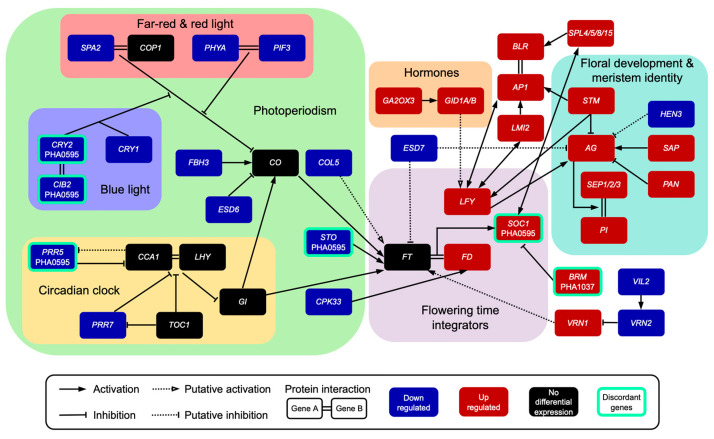
A hypothetical model for the regulatory network of the common bean’s floral transition under short-day length conditions. Down-regulated and up-regulated genes are indicated in blue and red, respectively. Discordant genes are squared in cyan and the name of the genotype in which is differentially expressed according to the color of its expression status. Gene interactions within the flowering pathways were inferred from *Arabidopsis* publications, most of them included in the database FLOR-ID [18]. Gene abbreviations are explained in Table 1. Evolutionarily conserved genes involved in *Arabidopsis* flowering pathways are listed in Tables S18–S20.

During the domestication of the temperate common bean, floral inductive signals replaced the mandatory photoperiod requirements of its wild tropical ancestor [34]. This control of the floral initiation timing has been studied widely in the LD *Arabidopsis* plant through the characterization of monogenic mutants and ‘natural variants’ that flower earlier or later than the wild type. These studies were performed extensively on the inductive LD conditions of *Arabidopsis*; however, under SD conditions they are still poorly understood [16,57]. Currently, it remains to be elucidated whether most of the components involved in the photoperiod flowering induction in the LD *Arabidopsis* plant are conserved in the SD common bean plant. Here, meristem expression profiles at the floral transition of two contrasting flowering behavior genotypes, PHA0595, a photoperiod-insensitive early-flowering cultivar, and PHA1037, a landrace with a strong photoperiod response similar to wild forms that behaved as an obligate SD plant, were analyzed to investigate central components of SD-dependent flowering pathways in the common bean. The findings regarding transcript abundance at defined developmental meristem stages provide the genetic bases for the formulation of biological hypotheses concerning the regulation of inflorescence development in the common bean and guide the design of further experiments that are required for functional validation. Based on these results, a hypothetical model for the regulatory networks involved in the common floral transition of the common bean is proposed and summarized in Figure 8.

The onset of flowering is characterized by a change in meristem identity from the vegetative to the inflorescence state, which is accompanied by alterations in the expression of key developmental genes in a complex network involving vernalization, autonomous, photoperiod, *GA*-dependent, and aging pathways. These flowering pathways can be both independent or linked and activate or inhibit floral transformation through several key floral integrators and floral meristem identity genes [12,14,16,50]. We studied expression patterns of *Arabidopsis* homologous floral integrator genes such as *LFY*, the flower meristem identity gene *AP1*, and *COL5* to identify additional marker genes for the vegetative and reproductive stages of the meristems of the common bean. Both *LFY* and *AP1* encode plant-specific transcription factors that have been shown to play key roles during flower development and flower meristem identity in diverse plant species that are required for the vegetative to reproductive growth transition [53,54,55]. It was found that the *LFY* and *AP1* homologues of the common bean were expressed strongly in IMs, and both are conserved regulators of the floral transition and suitable markers to distinguish IM from VM in the growing apex of beans (Figure 7).

This study revealed that among evolutionarily conserved DEGs involved in the flowering onset of the common bean, there were 15 genes related to the photoperiod pathway, of which 14 exhibited down-regulated expression at the IM-R6 flowering stage. Additionally, 10 genes were detected in both the PHA1037 and PHA0595 genotypes, and four genes were detected with a differential expression pattern depending on the genotype. In contrast, 12 out of the 13 genes involved in flower development and meristem identity were up-regulated at the IM-R6 flowering stage in both genotypes. Hence, expression results of both photoperiod-sensitive and photoperiod-insensitive genotypes point out that the floral transition under inductive SD conditions is promoted by the down-regulation of the photoperiodic-flowering genes and the upregulation of genes controlling floral meristem and flower organ identity (Table 1; Figure 8). In other crops, many floral development genes are photoperiod-regulated; for example, in apple with a low expression of photoperiod-related genes in the IM stage [58], in contrast with the upregulation in rose [59] and longan [60]. Our results indicate that the down-regulation of the photoperiodic-flowering genes is directly associated with the upregulation of inflorescence and flower formation related genes, which strongly suggests that photoperiod positively mediated the floral transition of the common bean. Furthermore, we postulate that these photoperiod pathway-related genes represent potential floral repressors that have to be inhibited to allow for the transition from VM to IM identity, thus triggering flowering in response to SD conditions.

An important network hub of the photoperiod flowering pathway is constituted by *CONSTANS* (*CO*)/*COL* genes, which integrate various environmental and internal signals [61]. In *Arabidopsis*, *CO* promotes flowering by activating the *FT* gene’s expression [62,63,64], and *FT* positively regulates *SOC1/AGL20* transcription [65] to promote flowering. The role of *Arabidopsis CO* as a LD floral activator is not shared in crops such as the common bean [35], and no differential expression of *CO* was detected in this study. Other legumes, such as *Medicago*, lack a *CO* homologue [37]. Interestingly, a homologue of the *Arabidopsis CO* gene, *COL5* (*Phvul.003G149000*), was highly up-regulated in the vegetative meristem before the transition to flowering (Table 1; Figure 8). The contrasting functionality of the common bean *COL5*, relative to the *Arabidopsis CO* [56,66], is similar to that observed for the common bean *COL2* [35], and it may be reminiscent of the demonstrated roles for *CO* in other species. For example, the rice *CO* orthologue *HEADING DATE 1* (*HD1*) has a dual role, acting as a floral activator or inhibitor under SD or LD conditions, respectively [67], and wheat *CO1* and *CO2* genes act as weak repressors of flowering and *FT* expression under LD and SD conditions [68]. Furthermore, in potato, *CO* represses expression of an *FT* homologue and delays tuberization, a process induced by SD lengths [69]. Although further work is necessary to determine its role during the transition to flowering in the common bean, we have identified *COL5* as an additional marker gene to discriminate between vegetative and reproductive stages, given that it was weakly expressed when flowering was induced in IM and up-regulated in VM before flowering.

Our study also revealed that other homologues of the *Arabidopsis* photoperiodic pathway involved in the positive regulation of flowering through activation of *CO* and *FT* expression were down-regulated in the inflorescence growth stage. Genes such as *PHYA2*, *CRYPTOCHROME 1* (*CRY1*) and *CRY2*, *PSEUDO-RESPONSE REGULATOR 5* (*PRR5*), *PRR7*, *SALT TOLERANCE* (*STO*), *CRY2-INTERACTING BHLH 2* (*CIB2*), *FLOWERING BHLH 3* (*FBH3*) *CALCIUM DEPENDENT PROTEIN KINASE 33* (*CPK33*), and *EARLY IN SHORT DAYS 6* (*ESD6*) [70,71,72,73,74,75,76,77] exhibited the lowest expression once the plant reached a reproductive stage, where they played a positive role in the regulation of flowering time (Table 1; Figure 8). Additionally, some of these genes did not show the same expression genotype-profile; *CRY2*, *CIB2*, *PRR5* and *STO* were down-regulated only in the IM of the temperate early-flowering PHA0595 cultivar (Table 1; Figure 8), suggesting that they may act as major day-length sensors in the common bean. Therefore, the dynamic changes and behavioral patterns of these genes may be the key reason for the no-sensitivity PHA0595 flowering period earlier than the sensitivity PHA1037.

In conclusion, this study constructed comparative transcriptomes from the tropical and temperate common bean cultivars and screened candidate genes related to flowering time. By considering the different flowering pathways, we have presented a model of the regulation of flowering transition in the common bean. Collectively, our study implies a divergence of the transcription networks controlled by clock-associated and light photoreceptor transcription factors of both *Arabidopsis* and common bean, which is a next step toward understanding the evolutionary development of the photoperiod network in common bean. The expression levels between two photoperiod-sensitive and photoperiod-insensitive genotypes, through VM to IM growth stages, illustrated the same trend, that is, down-regulation of the photoperiodic-flowering genes seems to be directly associated with the promotion of floral transition under inductive SD lengths through the upregulation of the floral integrators and flower development and meristem identity genes. We hypothesize that the dynamic changes and behavior patterns of photoperiod pathway genes with a discordant expression is the key reason for the no-sensitivity vs. sensitivity to flowering in common bean. These results provide further insight into the genetic control of flowering development in the common bean, as well as highlighting possible molecular breeding targets for shifting cultivation to higher latitudes. Further research is required to identify the regulatory pathways controlling flowering; however, future common bean breeding will eventually benefit from this knowledge.

## 4. Materials and Methods

### 4.1. Plant Growth and Tissue Collection

Plants of a nuña landrace PHA1037 and cultivar PHA0595 were grown in a growth chamber (20–25 °C, relative humidity 70–90%, 8-h day, and 16-h night SD photoperiod regime). PHA0595 is a Spanish improved line adapted to the LD photoperiod (>12-h day), which exhibits indeterminate erect growth habit type II [78] and early flowering (Figure 1A,C). PHA1037 is a LD photoperiod-sensitive nuña accession from Bolivia that possesses an indeterminate climbing growth habit type IV [78], and a strong photoperiod response similar to wild forms under LD conditions (Figure 1B,C). In both genotypes, vegetative axillary meristems (VM) and inflorescence meristems (IM) were collected. Samples at the VM stage (Figure 1D) were collected when common bean plants had >9 unfolded leaves (PHA0595 = 9, PHA1037 = 12), during the R5 pre-flowering phenological stage [39]. Samples at the IM stage (Figure 1E) were collected approximately 1 week later, during the R6 flowering stage [39]. For each stage and genotype, 18 different meristems from six independently random plants were manually dissected and pooled for each of the three biological replicates. All samples were collected in the morning, immediately frozen in liquid nitrogen, and stored at −80 °C until RNA-Seq was performed. 

### 4.2. RNA-Seq Analysis

Total RNA was extracted individually using TriFast Reagent (Peqlab, Erlangen, Germany) and the quality was assessed using NanoDrop 2000C (Thermo 116 Scientific, Wilmington, NC, USA), agarose gel electrophoresis, and Agilent 2100 BioAnalyzer (Agilent 117 Technologies, Santa Clara, CA, USA). Poly(A)-enriched cDNA libraries of VM and IM for both genotypes were generated using the NEBNext Ultra Directional RNA Library Prep Kit for Illumina (New England Biolabs, Ipswich, MA, USA), each with three biological replicates. The cDNA library insert size ranged 250–300 bp. The complete libraries were purified using the AMPure XP system (Beckman Coulter, Beverly, MA, USA) and qualified using the Agilent Bioanalyzer 2100 system. Finally, libraries were sequenced using an Illumina Hiseq 2500 platform (Illumina Inc., San Diego, CA, USA), and 150 bp paired-end reads were generated. The resulting raw short reads were deposited at NCBI Short Read Archive (SRA) under the BioProject accession code PRJNA854795 (https://www.ncbi.nlm.nih.gov/bioproject/PRJNA854795; accessed on 7 November 2022).

Sequencing reads were trimmed to remove adapters and low-quality bases using fastp version 0.20.1 software [79] with default options. Trimmed sequencing reads were then mapped against the *P. vulgaris* reference genome version 2.1 [80] using HISAT version 2.2.1 [81] with the option ‘very-sensitive’. To obtain the raw read table with the transcription levels of each gene in each sample, the feature Counts tool of the Subread suite version 2.0.1 [82] was used. For subsequent analyses, these transcript levels were normalized as transcripts per million (TPM) [83]. These TPM-normalized expression levels are provided in Table S1.

### 4.3. Identification of Differentially Expressed Genes

Prior to the differential expression analysis and to determine consistency between biological replicates, a Euclidean pairwise-distance analysis of all RNA-seq libraries was performed and plotted using Complex Heatmap [84] (Figure S1). A differential expression analysis was conducted using the local Wald test [85] implemented in the DESeq2 version 1.30.0 [86] to compare variance stabilizing transformation (VST)-normalized expression values of IM vs. VM for each genotype [87]. All genes with a false discovery rate (FDR) adjusted *p*-value [88] ≤0.05 were defined as differentially expressed genes (DEGs) and classified as up-regulated or down-regulated according to the sign of the logarithm of their fold-change values (Figure 3 and Figure S2; Tables S2–S5). Then, lists of up- and down-regulated genes in IM that are common to both genotypes were obtained (Tables S6–S7), as well as those DEGs that showed a discordant expression profile (that is, up-regulated in one genotype and down-regulated in the other) (Table S8).

### 4.4. Biological Processes and Pathways Analyses

To discover in which biological processes the DEGs are involved, an analysis of enriched GO [89] terms related to biological processes was performed using ClueGO version 2.5.8 [90]. This allows for the detection of those GO terms enriched within the up- and down-regulated gene sets (common to both genotypes) and within discordant genes. Because the functional annotation of *P. vulgaris* is poorly developed, the homologous genes of *Arabidopsis*, according to PhytoMine version 12 [91], were used (Tables S9–S11). The ClueGO enrichment test was used with the Bonferroni step down multiple testing correction [92] and a significance threshold of 0.005. GO term levels 7–10 were selected within the tree of ontological terms. GO terms with less than five genes or less than 5% DEGs were filtered out, and a kappa score of 0.4 was used (Tables S12–S14). The DEGs were classified according to the GO term groups to which they belong. To illustrate the possible overlap between sets of DEGs classified by the GO term groups, upset plots [93] for up- and down-regulated genes, and for discordant genes were constructed using ComplexHeatmap [84] (Figures S4–S6). *P. vulgaris* DEGs were also cross-checked with flowering pathway genes listed in the Flowering Interactive Database (FLOR-ID) [18] to identify which of them are homologous to the *Arabidopsis* flowering genes (Tables S12–S14). In addition, to evaluate the KEGG pathways [94] enriched within the up- and down-regulated gene sets (common to both genotypes) and within discordant genes, the enrichKEGG function of clusterProfiler package [95] was used together with the KEGG pathway database for the common bean. We selected as enriched KEGG pathways those whose corrected *p*-value for multiple testing was less than or equal to 0.05.

### 4.5. Evolutionary Conservation Analysis

With the aim of assessing the evolutionary conservation of genes related to flowering, DEGs common to both genotypes were analyzed to identify how many of them have homologous genes, using Phytozome version 12 [91], for *Arabidopsis* and seven species of the Fabidae clade [96], namely *Cucumis sativus*, *Fragaria vesca*, *G. max*, *Malus domestica*, *M. truncatula*, *Prunus persica* and *Trifolium pratense*. These evolutionarily conserved genes were also cross-checked with flowering pathway genes listed in FLOR-ID [18] to distinguish which of them are homologous to the previously described *Arabidopsis* flowering genes (Tables S18–S20).

### 4.6. Quantitative Real-Time PCR (qPCR) for Validation

To corroborate the findings of the RNA-seq analysis, each RNA from the VM and IM of each genotype was used to construct a cDNA library. The cDNA was synthesized from 100 ng of total RNA using M-MuLV reverse transcriptase (Fermentas Life Sciences, Hanover, MD, USA) with a mixture of random hexamer and oligo (dT) 18 primers. The SYBR Green PCR Master Mix kit (Applied Biosystems, Foster City, CA, USA) was used to perform qRT-PCR reactions in a 7300 Real-Time PCR System (Applied Biosystems, Foster City, CA, USA), according to the manufacturer’s instructions. All reactions were performed in duplicate at a volume of 10 μL, containing 1 μL of cDNA and 300 nM of each specific primer. The thermal cycles were set as follows: 95 °C for 10 min, 40 cycles at 95 °C for 15 s, and 60 °C for 1 min. At the end of each reaction, a melting curve analysis of amplification products was performed to confirm that only one PCR product was amplified and detected. Results were processed using the ∆∆Ct calculation method [97], expressed in arbitrary units and normalized by comparison to the housekeeping gene *UBIQUITIN* (*Phvul.001G193800*). Specific primer pairs for *Phvul.001G193800* (forward 5′-TTACATGCGCTCTTGGACTG-3′ and reverse 5′-CGAACACTTGGAGGCTTTTC-3′), *Phvul.003G281000* (*APETALA1*) (forward 5′-TTCGTACACGCAGAAACCAA-3′ and reverse 5′-TGGCTGTGGTAGCAAGAAAGA-3′), *Phvul.009G160900* (*LEAFY*) (forward 5′-GAGATCAAACGCCGCAATAG-3′ and reverse 5′-GGCTCCTCCGACAAACCT-3′), and *Phvul.003G149000* (*CONSTANS-LIKE 5*) (forward 5′-TCCCAGTCTCTCAGCCAAAG-3′ and reverse 5′-CGGTCCATTCCACACAACTG-3′), were used to perform qRT-PCR experiments. The qRT-PCR reactions were set up with three biological replications and three technical replicates per experiment.

## Figures and Tables

**Table 1:** Summary of the 66 evolutionarily conserved genes included in the FLOR-ID *Arabidopsis* flowering database.

Flowering and Flower Development Related Pathways	Up-Regulated	Down-Regulated	Discordant Genes
**Photoperiod**	*FD* (1)	*COL5* (1), *PHYA2* (1), *CRY1* (1), *PIF3* (1), *PRR7* (1), *EFS* (1), *FBH3* (1), *SPA2* (1), *CPK33* (1), *ESD6* (1)	*CRY2* (1, PHA1037), *CIB2* (1, PHA1037), *PRR5* (1, PHA1037), *STO* (1, PHA1037)
**Vernalization**	*FRL2* (1), *VRN1* (1), *POB1* (1)	*VIL2* (1), *VRN2* (1)	
**Autonomous**	*HTA11* (1)	*HUB1* (1), *ESD7* (1), *GLK1* (1)	*SKB1* (1, PHA0595), *BRM* (1, PHA1037), *HULK1* (1, PHA1037), *DCL1* (1, PHA1037)
**Hormones**	*GA2OX3* (1), *GID1* (2), *GAS4* (1), *ATH1* (2)	*HIPP3* (1), *GATA21* (2)	
**Aging**	*SPLs* (3)	*TPS1* (1)	
**Sugars**		*ADG1* (1), *PGM1* (1)	
**Flowering Integrators**	*LFY* (1), *GIN2* (1)		*SOC1* (1, PHA0595)
**Flower development and meristem identity**	*AP1* (1), *LMI2* (1), *BLR* (2), *SAP* (1), *SAP18* (1), *STM* (2), *PAN* (1), *AG* (2), *PI* (2), *ARF3* (2), *AGLs/SEP* (3), *STY1* (1)	*HEN3* (1)	

The number of differentially expressed genes homologous to each *Arabidopsis* flowering gene is shown between parentheses after each gene name. The discordant gene column also indicated the genotype in which the gene is up-regulated. Genes shown in bold are represented in Figure 8. Gene abbreviations and a detailed description of *Arabidopsis* and common bean gene IDs are provided in Table S21.

## Data Availability

The resulting raw short reads were deposited at NCBI Short Read Archive (SRA) under the BioProject accession code PRJNA854795 (https://www.ncbi.nlm.nih.gov/bioproject/PRJNA854795; accessed on 7 November 2022).

## Supplementary Materials

The following supporting information can be downloaded at: https://www.mdpi.com/article/10.3390/ijms232314783/s1.
