# Supplementary material for: Decoding Gene Expression Signatures Underlying Vegetative to Inflorescence Meristem Transition in the Common Bean
Source: Int J Mol Sci. 2022 Nov 26;23(23):14783. doi: 10.3390/ijms232314783 (PMC9739310; doi:10.3390/ijms232314783)
Supplement: Supplementary file 1 [file ijms-23-14783-s001.zip › Supplementary_Files/Supplementary Materials.pdf]

Supplementary materials

Supplementary Figures

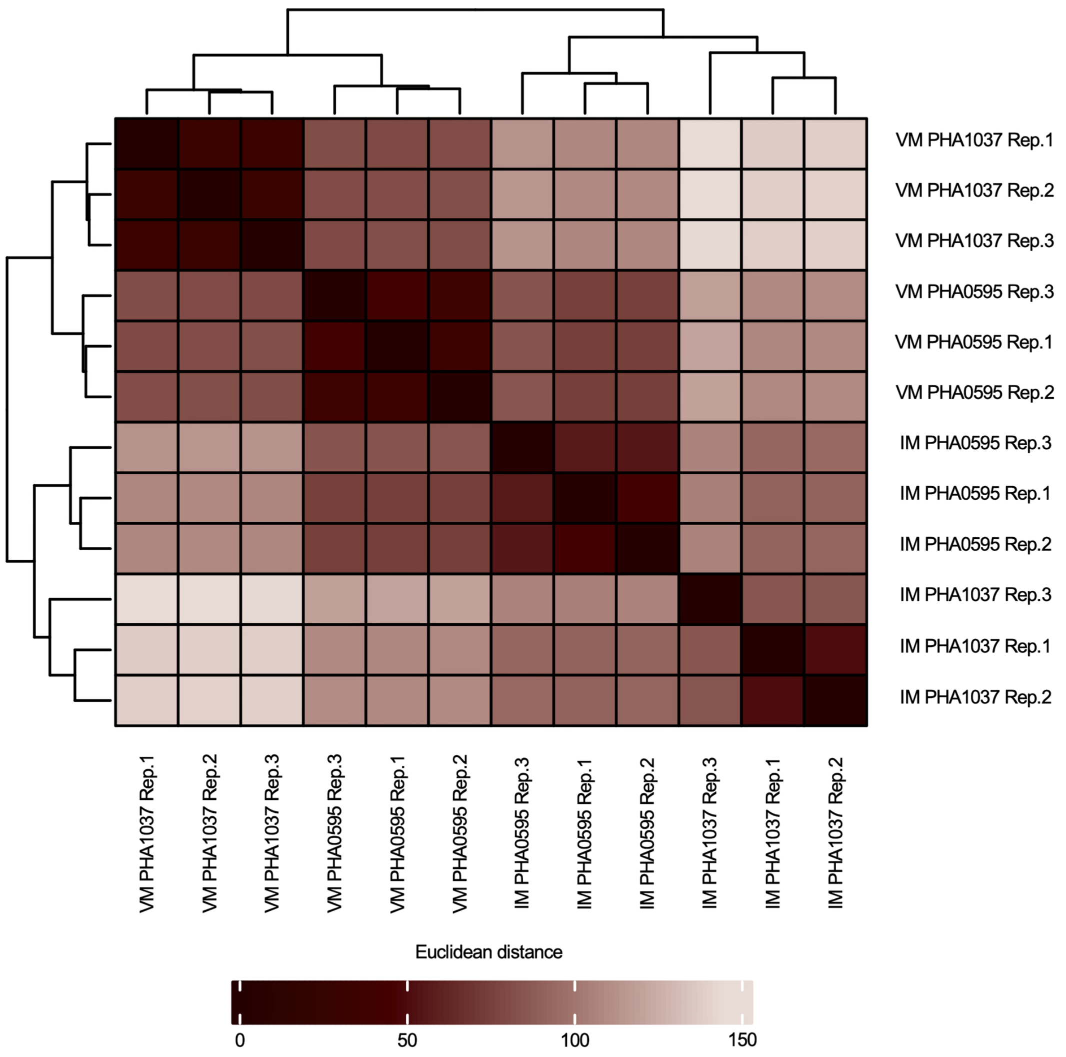

Figure S1. Euclidean distance matrix and hierarchical clustering of RNA-seq samples. IM: inflorescence meristem; VM: vegetative meristem; Rep.: biological replicate. Biological replicates clustered consistently. A greater overall dissimilarity (brighter in the color scale) between types of meristems was observed in PHA1037 than in PHA0595. The expression levels of each gene normalized as Transcripts Per Million (TPM) are available in Table S1.

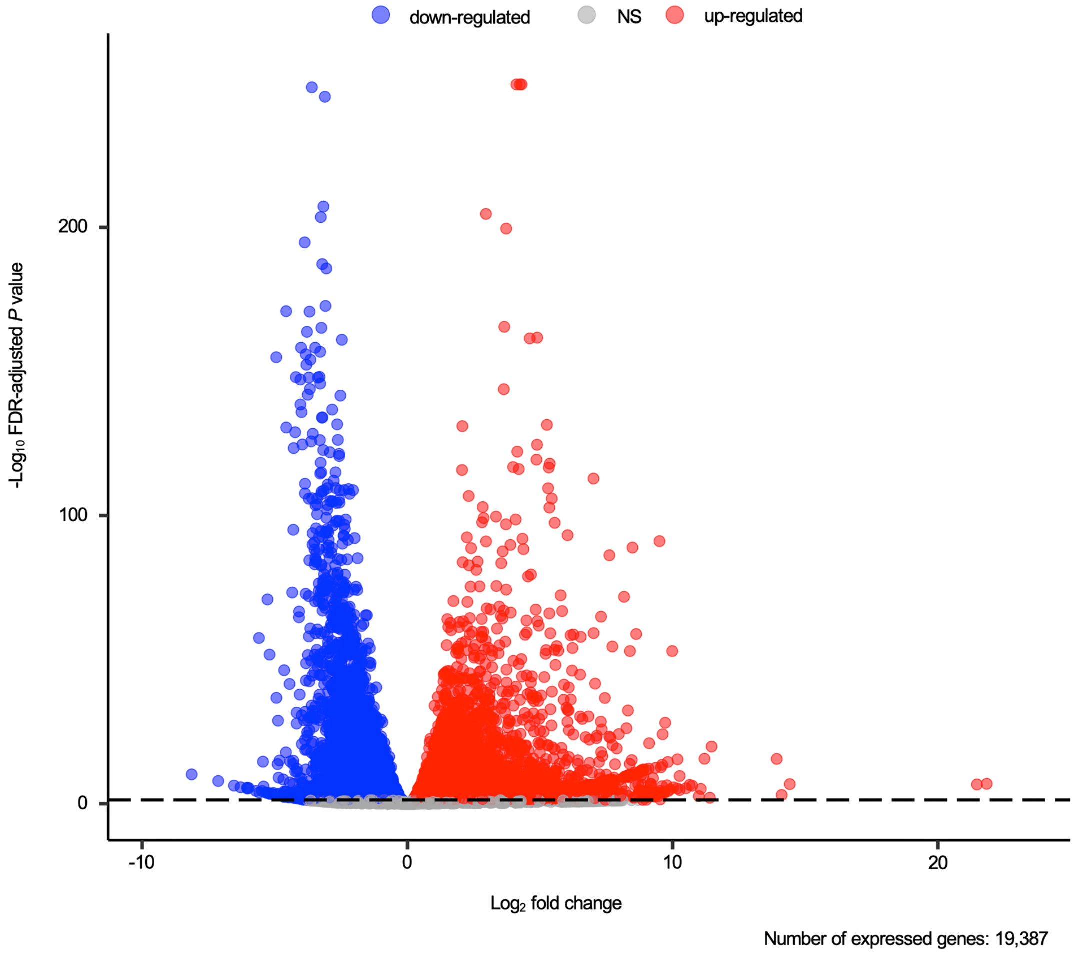

Figure S2. Volcano plot of genes differentially expressed in inflorescence meristem compared to vegetative meristem in PHA1037. Red dots correspond to up-regulated genes and blue dots to down-regulated genes. Gray dots are genes without significant expression differences. The horizontal axis shows the logarithm of the fold change and the vertical axis shows the minus logarithm of the p-value corrected for multiple testing (FDR). Details on these differentially expressed genes are given in Tables S2 and S3.

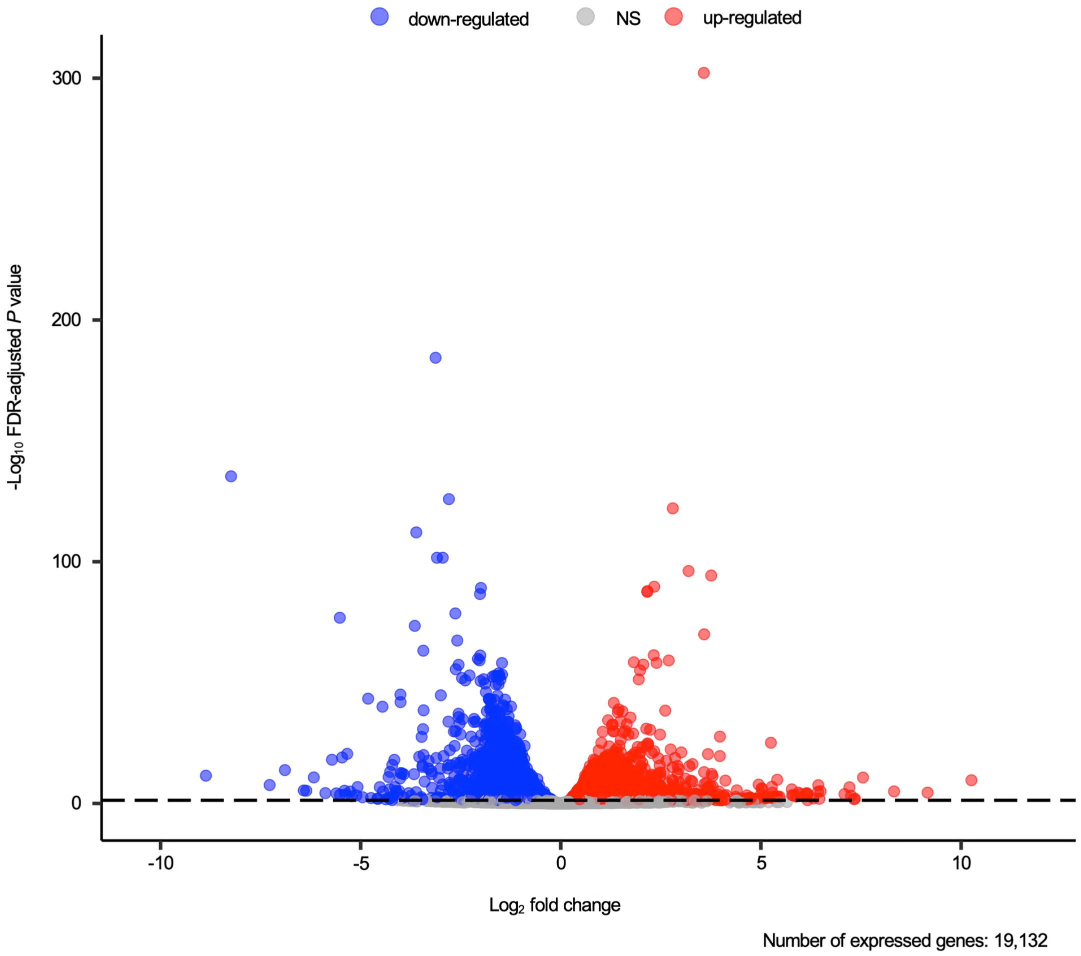

Figure S3. Volcano plot of genes differentially expressed in inflorescence meristem compared to vegetative meristem in PHA0595. Red dots correspond to up-regulated genes and blue dots to down-regulated genes. Gray dots are genes without significant expression differences. The horizontal axis shows the logarithm of the fold change and the vertical axis shows the minus logarithm of the p-value corrected for multiple testing (FDR). Details on these differentially expressed genes are given in Tables S4 and S5.

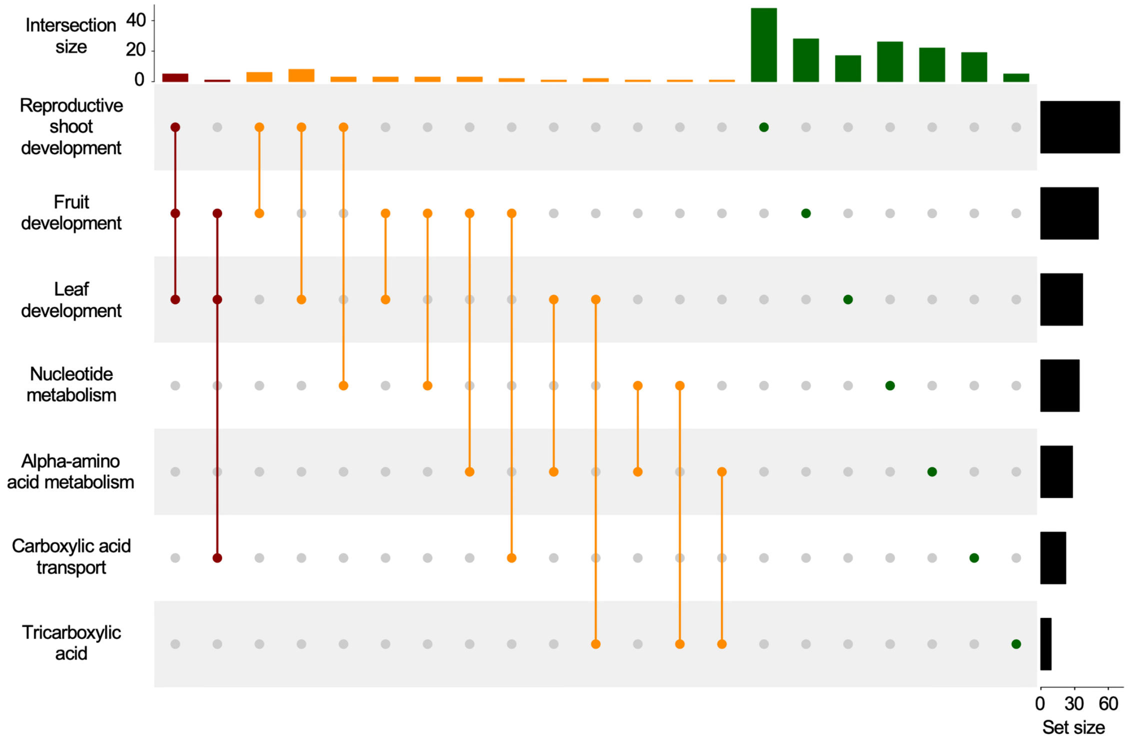

Figure S4. Upset plot of up-regulated genes common to PHA1037 and PHA0595, classified by the GO term group. At the top, the number of genes in each combination of GO term groups (intersection size) is shown, according to the combinations indicated at the bottom. On the right side, the number of genes composing each group of GO terms (set size) is shown. Genes belonging to a single category are highlighted in green, those belonging to two categories are highlighted in orange and those belonging to three categories are highlighted in dark red. Details of this ontological annotation can be found in Table S12.

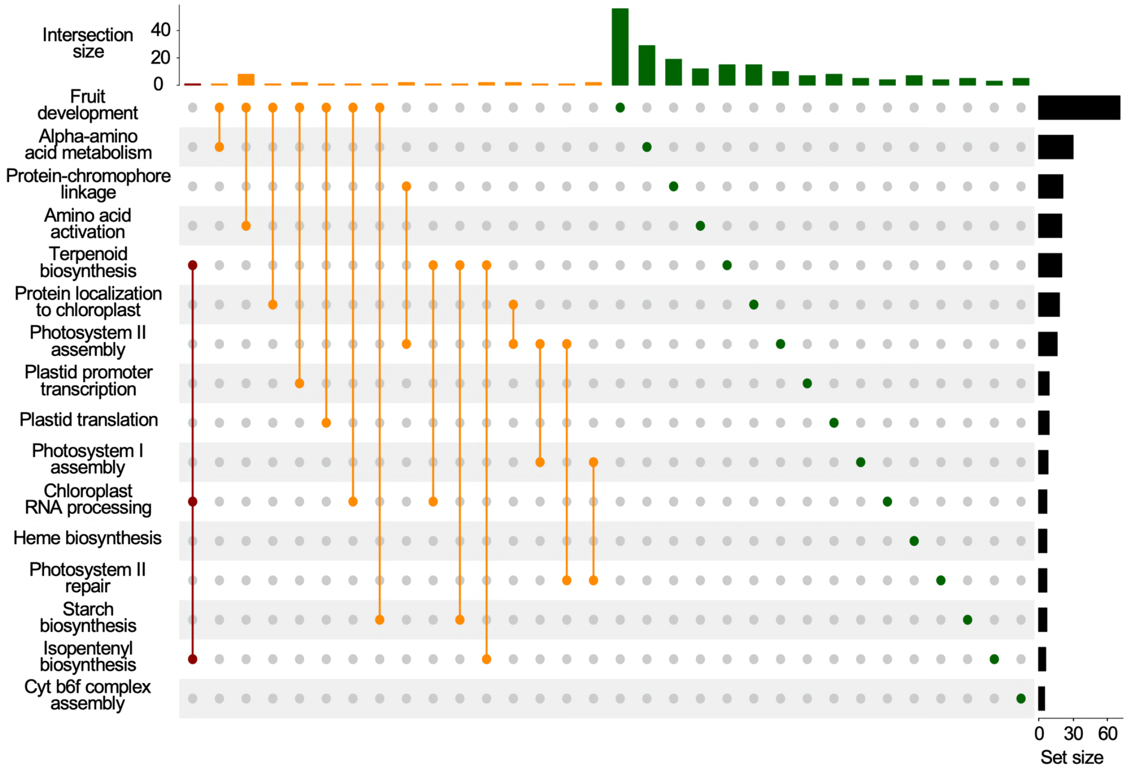

Figure S5. Upset plot of down-regulated genes common to PHA1037 and PHA0595, classified by the GO term group. At the top, the number of genes in each combination of GO term groups (intersection size) is shown, according to the combinations indicated at the bottom. On the right side, the number of genes composing each group of GO terms (set size) is shown. Genes belonging to a single category are highlighted in green, those belonging to two categories are highlighted in orange and those belonging to three categories are highlighted in dark red. Details of this ontological annotation can be found in Table S13.

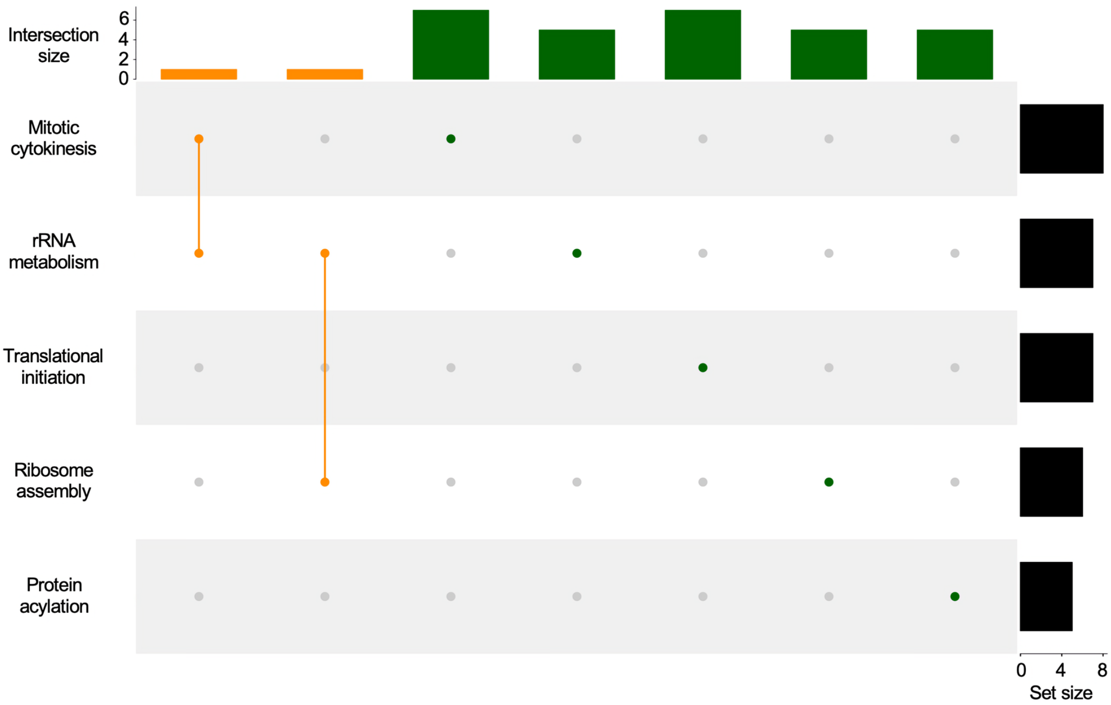

Figure S6. Upset plot of differentially expressed genes discordant between PHA1037 and PHA0595, classified by the GO term group. At the top, the number of genes in each combination of GO term groups (intersection size) is shown, according to the combinations indicated at the bottom. On the right side, the number of genes composing each group of GO terms (set size) is shown. Genes belonging to a single category are highlighted in green and those belonging to two categories are highlighted in orange. Details of this ontological annotation can be found in Table S14.

Supplementary Tables

Table S1. Gene expression in Transcripts Per Million (TPM). (TableS1.xlsx file).

Table S2. Gene up-regulated in inflorescence meristem compared to vegetative meristem in PHA1037. (Ta-bleS2-S8.xlsx file).

Table S3. Gene down-regulated in inflorescence meristem compared to vegetative meristem in PHA1037. (TableS2-S8.xlsx file).

Table S4. Gene up-regulated in inflorescence meristem compared to vegetative meristem in PHA0595. (TableS2-S8.xlsx file).

Table S5. Gene down-regulated in inflorescence meristem compared to vegetative meristem in PHA0595. (Ta-bleS2-S8.xlsx file).

Table S6. Gene up-regulated in inflorescence meristem compared to vegetative meristem in both PHA1037 and PHA0595. (TableS2-S8.xlsx file).

Table S7. Gene down-regulated in inflorescence meristem compared to vegetative meristem in both PHA1037 and PHA0595. (TableS2-S8.xlsx file).

Table S8. Differentially expressed genes between inflorescence and vegetative meristems with opposite expression patterns in PHA1037 and PHA0595. (TableS2-S8.xlsx file).

Table S9. *Arabidopsis* thaliana homologous genes for the up-regulated gene set. (TableS9-S11.xlsx file).

Table S10. *Arabidopsis* thaliana homologous genes for the down-regulated gene set. (TableS9-S11.xlsx file).

Table S11. *Arabidopsis* thaliana homologous genes for the discordant gene set. (TableS9-S11.xlsx file).

Table S12. GO Biological Process terms enriched for the up-regulated gene set. (TableS12-S14.xlsx file).

Table S13. GO Biological Process terms enriched for the down-regulated gene set. (TableS12-S14.xlsx file).

Table S14. GO Biological Process terms enriched for the discordant gene set. (TableS12-S14.xlsx file).

Table S15. KEGG pathways enriched for the up-regulated gene set. (TableS15-S17.xlsx file).

Table S16. KEGG pathways enriched for the down-regulated gene set. (TableS15-S17.xlsx file).

Table S17. KEGG pathways enriched for the discordant gene set. (TableS15-S17.xlsx file).

Table S18. Homologous genes of the common up-regulated genes. (TableS18-S20.xlsx file).

Table S19. Homologous genes of the common down-regulated genes. (TableS18-S20.xlsx file).

Table S20. Homologous genes of the discordant genes. (TableS18-S20.xlsx file).

Table S21. Common bean evolutionarily con-served genes included in the FLOR-ID *Arabidopsis* flowering database. (TableS21.xlsx file).
